# NSUNs-driven dysregulation: the next frontier in targeted cancer therapy?

**DOI:** 10.1038/s41419-025-08269-6

**Published:** 2025-12-18

**Authors:** Yingqi Zhao, Yuying Zhang, Haonan Zhou, Lei Zhang, Jinyu Guo, Yue Tan, Ting Wu, Yi Peng, Ying Che, Zhanlei Pei, Jun Li, Boshi Fu, Minjie Wei, Gang Wu, Xiaoyun Hu, Huizhe Wu

**Affiliations:** 1 Department of Pharmacology, School of Pharmacy, China Medical University, Shenyang, PR China; 2 Liaoning Key Laboratory of molecular targeted anti-tumor drug development and evaluation; Liaoning Cancer immune peptide drug Engineering Technology Research Center; China Medical University, Shenyang, PR China; 3 Hepatobiliary Surgery Department, First Hospital of China Medical University, Shenyang, Liaoning Province PR China; 4 Shenyang Kangwei Medical Laboratory Analysis Co. LTD, Liaoning Province Shenyang, PR China; 5 Scientific experimental center, School of Pharmacy, China Medical University, Shenyang, PR China

**Keywords:** Tumour biomarkers, Epigenomics

## Abstract

Epitranscriptomic modifications represent a fundamental regulatory layer in cancer biology, with RNA methylation emerging as a pivotal mechanism governing transcriptomic dynamics. Among these, 5-methylcytosine (m^5^C) RNA methylation—a ubiquitous and conserved epitranscriptomic mark—has been identified across diverse RNA species, including mRNAs, rRNAs, tRNAs, and mitochondrial RNAs. Notably, the RNA m^5^C “writers”—enzymes responsible for installing this modification onto target RNAs—have emerged as central regulators of tumorigenesis, with NSUN (NOP2/Sun RNA methyltransferase) proteins playing a particularly pivotal role. We synthesize current knowledge of the cellular localization, substrate specificity, and biological functions of m^5^C-modifying enzymes, focusing predominantly on the NSUN family in the cancer context. We first dissect the spatiotemporal regulation patterns of NSUN proteins—from their nuclear roles in pre-mRNA processing to cytoplasmic functions in mRNA decay and translation—and their conserved methyltransferase domains that dictate target RNA recognition. This review further explores the molecular mechanisms by which NSUN proteins govern tumor progression, metastasis, and therapeutic responses, emphasizing their dual roles in both initiating oncogenic programs and maintaining cancer cell plasticity. Finally, we discuss the translational implications of targeting NSUN-mediated m^5^C pathways, highlighting small-molecule inhibitors designed against NSUN substrate specificity, combinatorial strategies with conventional chemotherapy or immunotherapy, and the promise of epitranscriptomic diagnostics and prognostic based on NSUN expression signatures. By positioning NSUN proteins as integral nodes in the RNA epigenomic network, this synthesis not only deepens our understanding of cancer pathogenesis but also identifies novel epitranscriptomic targets for precision oncology.

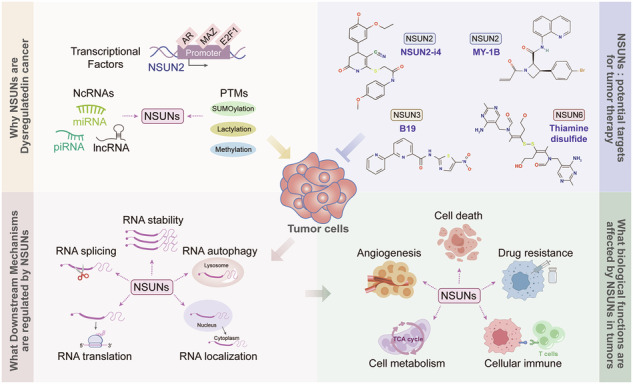

## Facts

- NSUNs, key methyltransferases of RNA m^5^C modification, can mediate multiple biological functions such as tumor death, angiogenesis, metabolic regulation, immune response, and drug resistance.
- NSUNs participate in regulating RNA stability, translation efficiency, and intracellular localization by catalyzing m^5^C modifications of various RNAs such as mRNA, tRNA, rRNA, and lncRNA.
- As potential biomarkers for tumor diagnosis and prognosis evaluation, targeted inhibition of NSUNs expression and function has become a hot topic in current tumor drug development.
- At present, research on NSUNs as drug development targets still has significant limitations, and there is a large research gap in the development of new intervention strategies, posing severe challenges to targeted drug research.

## Open questions

- How to construct a composite biomarker that integrates m⁵C modification profiles with tumor genetic characteristics to advance non-invasive and precise diagnosis and treatment?
- Do different members of the NSUNs family exhibit an elaborate spatiotemporal coordinated regulatory mechanism?
- The mechanism by which crosstalk between non-coding RNAs and NSUNs affects tumor cell fate remains largely unexplored.
- Can novel targeted drugs be developed by disrupting NSUNs-RNA interactions or using protein degradation technologies?

## Introduction

Epitranscriptomics has remained a central focus in life sciences since the post-genomic era [1, 2], following the discovery of N6-methyladenosine (m^6^A) in mRNAs, over 170 RNA modifications have been identified, with m^5^C emerging as a highly prevalent epitranscriptomic mark in various RNA species [3–6]. The m^5^C modification was first discovered in DNA in 1950 by Wyatt GR [7]. The presence of m^5^C in RNA reveals a mechanism analogous to that of DNA m^5^C, where a methyl group—donated by S-adenosylmethionine (SAM)—is attached to the fifth carbon of the cytosine base to form m^5^C [8, 9]. In 1974, the first eukaryotic m^5^C modification was identified in *Escherichia coli* and rat liver tRNA [10, 11]. Advancements in bisulfite sequencing (BS-seq) and methylated RNA immunoprecipitation sequencing (MeRIP-seq) have facilitated the precise mapping of RNA m^5^C modifications, establishing a foundation for in-depth RNA studies [12–14].

Currently, RNA m^5^C modifications are implicated in a range of cancers and have been detected across various RNA species, such as mRNAs, tRNAs, rRNAs, lncRNAs, scaRNAs, miRNAs, and mt-RNAs [15–18]. This reversible modification modulates RNA stability, translation efficiency, splicing, and export, regulated by the concerted actions of RNA cytosine methyltransferases (RCMTs), demethylases, and reader proteins [19, 20]. A variety of m^5^C RCMTs have been identified, including members of the NOL1/NOP2/SUN domain family and the DNA methyltransferase (DNMT) family [21]. DNMTs were initially thought to catalyze m^5^C modification in DNA [22]; however, subsequent studies revealed that only DNMT2 is capable of catalyzing m^5^C modification in RNA, and it stabilizes tRNA and mRNA through this modification [23–26].

**Fig. 1:**
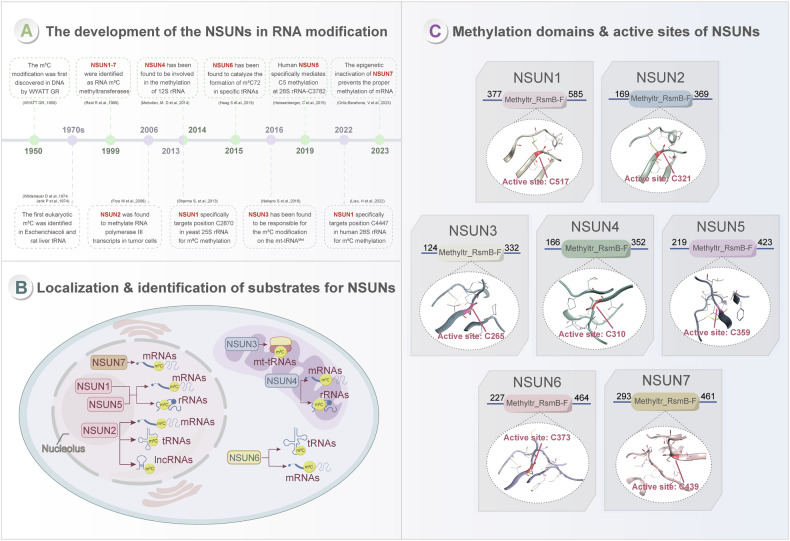
The subcellular localization and structural domains of NSUN proteins. **A** Timeline of NSUNs family in RNA m⁵C modification research. This timeline outlines key milestones in research on NSUNs-mediated 5-methylcytosine (m⁵C) modification of RNA, spanning from the first identification of m⁵C in DNA (1950) to the successive characterization of NSUN1–7 in m⁵C modification of diverse RNAs (e.g., tRNA, rRNA, mRNA) [7, 10, 11, 15, 21, 41, 44, 46, 49–51, 54]. **B** The different cellular localizations of NSUN proteins and identified their substrates. Among them, NSUN1, NSUN2, and NSUN5 are mainly located in the nucleus, NSUN3 and NSUN4 are primarily found in mitochondria, and NSUN6 is mainly located in the cytoplasm [37, 39, 43, 46, 48, 50–52, 54]. **C** The key methylation domains and active sites of NSUN proteins. These have separately displayed the methylation domains of different NSUNs based on the SMART database (https://smart.embl.de/), while marking the active sites of each NSUN protein in pink.

Compared to the DNMT family, the NSUN family—a key subclass of m^5^C “writers”—is the primary mediator of RNA m^5^C modification. In 1999, Reid et al. identified NSUN1-7 as RNA m^5^C methyltransferases, based on the amino acid sequence of Fmu—a well-characterized RNA m^5^C methyltransferase [21]. Subsequently, Fig. 1A systematically presents the research development timeline of the NSUNs family involved in RNA m⁵C modification, clearly marking the key nodes at which different NSUN members were gradually reported to possess the catalytic function of RNA m⁵C modification. The timeline traces the scientific journey from the initial identification of m⁵C in DNA in 1950 to the subsequent characterization of NSUN1-7 and their specific functions in depositing m^5^C modifications across various RNA species, including tRNAs, rRNAs, and mRNAs. NSUN proteins contribute to oncogenesis through RNA m^5^C modifications, with their involvement confirmed in various cancers [27–31]. For instance, NSUN2 has been identified as a marker and novel target for nasopharyngeal carcinoma (NPC) and prostate cancer (PCa) [30, 31]. Subsequently, the functions and mechanisms of NSUNs in tumors have been gradually elucidated [32, 33]. Such as, Wang et al. showed that NSUN2 promoted cervical cancer (CC) cell migration and invasion by recruiting YBX1 to stabilize *KRT13* mRNA [32]. Zhang et al. demonstrated that NSUN2, overexpressed in breast cancer, collaborated with YBX1 to bind *HGH1* mRNA via m^5^C methylation, enhancing its stability and promoting breast cancer (BC) cell migration and invasion [33]. These mainly regulate RNA fate to mediate biological functions and participate in tumor progression.

Therefore, this review summarizes the functional mechanisms by which NSUNs regulate RNA metabolism, while critically evaluating the subcellular localization and structural properties of NSUN proteins, emphasizing their roles as key enzymes in RNA m^5^C modification and their involvement in tumor progression. It consolidates the current understanding of m^5^C regulators, focusing on NSUN-mediated RNA m^5^C modifications within the context of tumorigenesis, drug resistance, and cancer immunotherapy. Additionally, the review explores ongoing research on targeted NSUN inhibitors, aiming to identify novel tumor biomarkers and further the development of precision medicine for personalized cancer therapies.

## Overview of the NSUN protein: subcellular localization and structural domains

The NOP1/NOP2/SUN domain family includes proteins that bind S-adenosylmethionine, including NSUN1, NSUN2, NSUN3, NSUN4, NSUN5, NSUN6, and NSUN7 [34, 35]. These proteins utilize a shared catalytic mechanism for m^5^C methylation, where covalent intermediates are formed between two catalytic cysteine residues at the active site and cytosine in RNA. This interaction activates the electron-deficient pyrimidine ring, facilitating a nucleophilic attack on the methyl group of SAM at the C5 position of cytosine [36, 37]. Nevertheless, variations in their subcellular localization lead to distinct catalytic substrates. This section examines the subcellular distribution of NSUN proteins, their respective substrates, and the specific methylation domains and catalytic sites involved, offering insights into their structural and functional roles in tumor-related m^5^C methylation (illustrated in Fig. 1B and C).

Nucleolar protein 2 (NOP2), also known as NSUN1, is an RNA methyltransferase composed of 812 amino acids. Initially recognized as the proliferating cell nuclear antigen p120, NOP2 is primarily localized in the nucleolus [38, 39]. Subsequent studies revealed that NSUN1 specifically methylates rRNAs, with C2870 in yeast *25S* rRNA in 2013 and C4447 in human *28S* rRNA in 2022 serving as key targets [15, 40, 41]; while Cys517 functions as the principal nucleophilic active site and Cys459 as the release site [42]. NSUN2, a 767-amino acid protein predominantly localized in the nucleus, interacts with a broad array of substrates, including rRNA, tRNA, mRNA, and ncRNA [43]. As early as 2006, NSUN2 was found to methylate RNA polymerase III transcripts in tumor cells, mediating Myc-induced cell proliferation and growth [44]; The active nucleophile Cys321 catalyzes m^5^C methylation, with Cys271 serving as the release site. Through these sites, NSUN2 modulates gene m^5^C activity, playing a substantial role in cancer progression [45]. Similarly, NSUN5, a 429-amino-acid protein primarily localized in the nucleolus, catalyzes m^5^C methylation at position C3782 in *28S* rRNA. The catalytic mechanism depends critically on the principal nucleophilic active site Cys359, which is essential for this modification [46]. In 2015, researchers identified that human NSUN5/yeast Rcm1 specifically mediates C5 methylation at C2278 in *25S* rRNA(yeast) [47]. Currently, the precise subcellular localization of NSUN7 remains unclear, though it has been detected in the nucleus, where it promoted PGC-1α-mediated transcription [48]. NSUN7 consists of 718 amino acids. Its active site, Cys439, functions as the primary nucleophile for m^5^C methylation, and its substrate is *CCDC9B* mRNA [49].

Cytoplasm, a key cellular compartment, houses various proteins. NSUN6, mainly confined to the cytoplasm, consists of 469 amino acids and interacts with a broad spectrum of substrates, including The formation of C72 in specific *tRNA* [50]; The Cys373 residue, located within the enzyme’s active site, acts as the primary nucleophile in catalyzing m^5^C methylation [50].

In addition to the nucleus and cytoplasm, mitochondria represent another essential subcellular compartment for NSUN proteins, specifically NSUN3 and NSUN4. Composed of 340 amino acids, NSUN3 mediates the m^5^C methylation of mt-tRNA within mitochondria, specifically catalyzing methylation at position 34 of *mt-tRNA* (Met), thereby initiating f^5^C formation [51, 52]. The active site Cys265 acts as the primary nucleophile responsible for this modification [53]. NSUN4, primarily localized in mitochondria [54], consists of 384 amino acids and targets rRNA through the MTERF4-NSUN4 complex [55]. In this context, Cys310 in NSUN4 serves as a catalytic nucleophile, whereas Cys258 eases product release from the covalent enzyme-RNA intermediate [56].

Together, existing studies indicate that NSUN proteins modulate m^5^C methylation, with cellular localization, methylation domains, and catalytic sites being integral to their functions. Further exploration of these domains and sites is necessary to elucidate the mechanisms by which these proteins participate in oncogenesis from a structural biology standpoint. Such insights may enable the development of targeted therapies that address specific active sites for cancer treatment.

## NSUNs mediate tumor progression

**Fig. 2:**
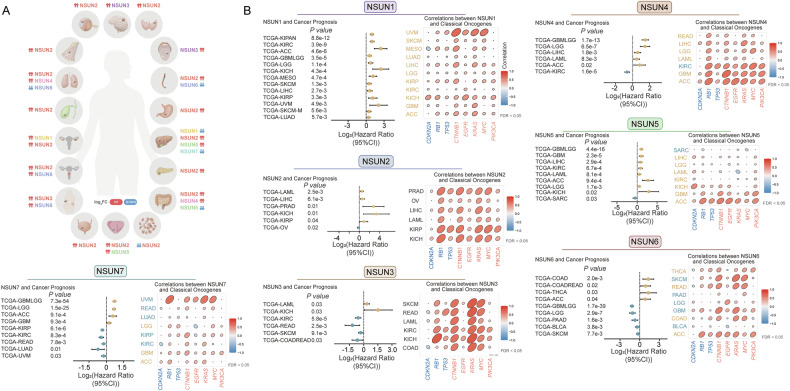
NSUNs mediating tumor progression. **A** Abnormal levels of NSUN proteins in various cancers. The red font indicates the up-regulation of the expression level in cancers, while the blue font indicates the down-regulation. **B** Bioinformatics-based approach to analyze the key role of other NSUNs in cancer prognostic and cancer-related genes (https://shengxin.ren/). The forest plot (left) and gene association bubble plot (right) were used to analyze the prognostic association of the NSUN family with cancers, as well as their correlation with classical cancer-associated genes. Yellow represents high-risk cancers, and blue represents low-risk cancers. Abbreviations: KIPAN kidney pancreas, KIRC kidney renal clear cell carcinoma, KIRP kidney renal papillary cell carcinoma, GBMLGG glioblastoma multiforme and lower—grade glioma, LGG lower—grade glioma, ACC adrenocortical carcinoma, KICH kidney chromophobe, MESO Mesothelioma, LUSC lung squamous cell carcinoma, LUAD lung adenocarcinoma, SKCM skin cutaneous melanoma, UCEC uterine corpus endometrial carcinoma, UVM uveal melanoma, SARC sarcoma, LIHC liver hepatocellular carcinoma, STAD stomach adenocarcinoma, AML acute myeloid leukemia, COAD colon adenocarcinoma, READ rectum adenocarcinoma, THCA thyroid carcinoma, PAAD pancreatic adenocarcinoma, BLCA bladder urothelial carcinoma, BRCA breast invasive carcinoma, OV ovarian serous cystadenocarcinoma.

NSUN proteins play a significant role in tumor progression and are closely associated with cancer prognosis (Table 1 and Fig. 2A), making them potential targets for cancer diagnosis and therapy [57–61]. This review synthesizes the current understanding of NSUNs—well-characterized m^5^C “writers”—in tumorigenesis, as well as the role of their mediated RNA m^5^C modification pathways as biomarkers across diverse cancer types. For example, Sun et al. identified NSUN2 as a prognostic biomarker in hepatocellular carcinoma (HCC) [58], while Wang et al. demonstrated its dual relevance as a prognostic and therapeutic marker in EGFR-TKI-resistant non-small cell lung cancer (NSCLC) [60]. Furthermore, Chen et al. highlighted the potential of NSUN2 as a diagnostic marker and therapeutic target in urothelial carcinoma of the bladder (UCB) [61]. Bioinformatics analyses have also been performed to explore the roles of other NSUNs in cancer prognosis and emphasized the association between NSUNs and very well-known genes involved in cancer(Fig. 2B). The forest plot and gene association bubble plot respectively analyze the association between NSUN family genes and cancer prognosis, as well as their correlation with classical cancer-associated oncogenes. These results highlight the crucial role of NSUNs as prognostic biomarkers in cancer. NSUNs may work together with classical oncogenes and suppressor genes, promoting cancer progression. This finding provides a theoretical basis for further study of molecular mechanisms. Furthermore, the strategy of integrating the “forest plot (prognostic analysis)” and “bubble plot (molecular correlation analysis)” to construct a “prognosis-molecule axis” further confirms that the NSUN family is a key participant in cancer prognostic regulation and oncogenic networks, providing important references for in-depth interpretation of its specific mechanisms of action and biological functions. Taken together, these results suggest that NSUNs play a critical role in tumorigenesis and may serve as important biomarkers for both cancer diagnosis and treatment.

**Table 1:** The role of NSUNs in different tumors.

NSUNs	Cancer type	Expression	Prognosis	Target genes or pathways	Biological function	Source of difference	References
NSUN1	HGSOC	Up-regulated	Unfavorable	*RAPGEF4*	Promote cell proliferation, migration, and invasion.	GSE10971, HGSOC patient tissue and TCGA	[126]
NSUN2	PC	Up-regulated	Unfavorable	*TIAM2*	Promote cell proliferation, migration, and invasion.	TCGA and GTEx GSE62452 PC patient tissues	[86]
	GC	Up-regulated	Unfavorable	*NTN1*	Promotes the migration, invasion, and NI potential.	GC patient tissues	[73]
	GC	Up-regulated	Unfavorable	*FOXC2*	Promote cell proliferation, migration, and invasion.	GC patient tissues	[74]
	GC	Up-regulated	Unfavorable	*P57*	Promote cell proliferation.	TCGA and GC patient tissues	[89]
	GC	Up-regulated	Unfavorable	*NR 033928*	Promote the expression of GLS and GC proliferation.	TCGA and GC patient tissues	[96]
	CC	Up-regulated	Unfavorable	*KRT13*	Promote cancer invasion and migration.	GSE17025 and GSE106191, GSE12470 and GSE66957, GSE138080 and GSE36514, UALCAN website, oncomine, cBioPortal, CC patient tissues	[32]
	CC	Up-regulated	Unfavorable	*LRRC8A*	Promote tumor growth and metastasis.	GSE63514, GSE52904, Oncomine CC patient tissues	[111]
	BC	Up-regulated	Unfavorable	*HGH1*	Promote tumor growth and migration.	TCGA and BC patient tissues	[33]
	Pca	Up-regulated	Unfavorable	*AR*	Activates positive feedback loop to promote prostate cancer.	TCGA High-risk PCa patients tissues	[31]
	ESCC	Up-regulated	Unfavorable	*NMR* (ERK signaling)	Promote tumor cell migration and invasion, inhibit cisplatin-induced apoptosis.	/	[59]
	ESCC	Up-regulated	Unfavorable	*GRB2*	Promote cell proliferation.	ESCC patient tissues and GSE53625	[68]
	RB	Up-regulated	Unfavorable	*PFAS*	RB growth and aggressiveness.	RB patient tissues and GSE111168	[66]
	EC	Up-regulated	Unfavorable	*SLC7A11*	Increase lipid peroxidation and ferroptosis, promote tumor growth.	EC patient tissues, TCGA and CPTAC	[80]
	UCB	Up-regulated	Unfavorable	*RABL6* and *TK1*	Enhances proliferation and invasion.	UCB patient tissues and TCGA	[97]
	CRC	Up-regulated	Unfavorable	*SKIL*	Promoted CRC malignancy.	TCGA, GSE20916, GSE33113, GSE8671 and CRC patient tissues	[27]
	CRC	Up-regulated	Unfavorable	*ENO1*	Promote reprogramming of glucose metabolism.	TCGA and CRC patient tissues	[83]
	OV	Up-regulated	Unfavorable	*E2F1*	Promoted malignancy.	TCGA, GSE19071, GSE18520, GSE27651and OV patient tissues	[69]
	AML	Up-regulated	Unfavorable	*PHGDH, SHMT2*	Affect serine metabolism.	TCGA, BloodSpot database and AML patients tissues	[125]
	HCC	Up-regulated	Unfavorable	*H19*	Promote the proliferation, migration, and invasion.	HCC cell lines	[58]
	NSCLC	Up-regulated	Unfavorable	*NRF2*	Increases NSCLC ferroptosis-resistance capacity.Promote cell proliferation.	TCGA,GSE33532, GSE31210 and NSCLC tumor tissues	[113]
	NSCLC	Up-regulated	Unfavorable	*QSOX1*	Promotes gefitinib resistance.	NSCLC patient tissues	[60]
	CCA	Up-regulated	Unfavorable	*NKILA*	Enhances proliferation and invasion.	TCGA CCA patient tissues	[91]
	OS	Up-regulated	Unfavorable	*FABP5*	Promote fatty acid metabolism.	GSE126209, GSE99671 and OS patient tissues	[124]
	GBM	Up-regulated	Unfavorable	*LINC00324*	Promote cell proliferation, migration and angiogenesis.	UALCAN	[93]
	HCC(HBV)	Up-regulated	Unfavorable	*HBV*	Regulates HBV infection and replication.	/	[90]
	ccRCC	Up-regulated	Unfavorable	*NEO1*	Promote glycolysis and histone lactylation.	/	[122]
	GC	Up-regulated	Unfavorable	*ATG9A*	Promotes 5-Fluorouracil resistance.	/	[132]
	HCC	Up-regulated	Unfavorable	*SOAT2*	Promote immune evasion.	GSE25599, GSE105130,GSE112705,GSE135631,GSE164359,GSE202069,GSE202853,GSE214846, GSE216613 and HCC patient tissues	[138]
	OS	Up-regulated	Unfavorable	*LINC01082*	Promote cell proliferation, migration.	GEPIA and OS patient tissue	[92]
	HCC	Up-regulated	Unfavorable	*MALAT1*	Promotes sorafenib resistance.	HCC patient tissues	[95]
NSUN3	HNSCC	Up-regulated	Unfavorable	*tRNA-C34*	Enhances mitochondrial translation rates and OXPHOS.	HNSCC patient tissues	[16]
NSUN4	CRC	Up-regulated	Unfavorable	*NXPH4*	Promote the proliferation and growth.	TCGA, GSE37182, GSE83889 and CRC patient tissues	[107]
	GBM	Up-regulated	Unfavorable	*CDC42*	Promote cell proliferation.	UCSC-XENA and GBM patient tissues	[87]
NSUN5	PCa	Up-regulated	Unfavorable	*ACC1*	Promote cell proliferation.	TCGA and PCa patient tissues	[85]
	HCC	Up-regulated	Unfavorable		Promote the proliferation and migration.	TCGA and HCC patient tissues	[103]
	COAD	Down-regulated	Favorable	*GPX4* (cGAS-STING signaling)	Regulates cancer immunotherapy.	TCGA and GSE17538	[123]
	GBM	Up-regulated	Unfavorable	*28S* rRNA	Promote overall protein translation and synthesis.	TCGA and GBM patient tissues	[102]
NSUN6	CC	Up-regulated	Unfavorable	*NDRG1*	Promotes radioresistance.	CC patient tissues	[135]
	ESCC	Down-regulated	Favorable	*CDH1*	Inhibit proliferation, migration, and invasion.	TCGA and ESCC patient tissues	[104]
	LUNG	Down-regulated	Favorable	*NM23-H1*	Inhibit proliferation and invasion and EMT.	TCGA	[62]
	OS	Up-regulated	Unfavorable	*EEF1A2* (Akt/mTOR signaling)	Promote proliferation, migration, and invasion.	GSE126209	[63]
NSUN7	HCC	Down-regulated	Favorable	*CCDC9B*	Inhibit the poor prognosis of tumors.	TCGA	[49]

### Why NSUNs are dysregulated in cancer

**Fig. 3:**
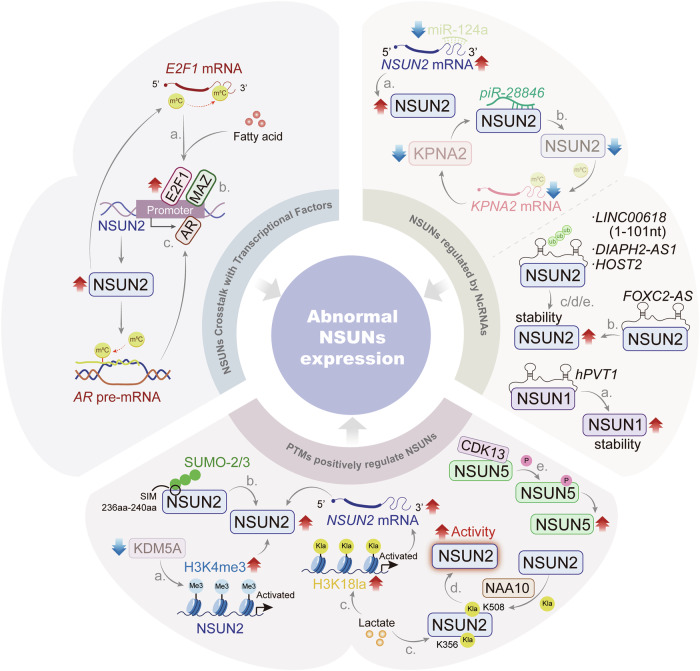
Upstream regulatory mechanisms of NSUNs. Upstream regulatory mechanisms of NSUNs, including encompassing transcriptional factor modulation, ncRNAs recruitment regulation and post-translational protein modifications [31, 67–85]. (Detailed information is available in the main text.).

Previous studies have established that abnormal NSUN expression in tumors promotes various tumorigenic processes [62–66]. This section examines the regulatory mechanisms responsible for NSUN dysregulation, including modulation by transcription factors, ncRNA-mediated recruitment, and post-translational modifications, as illustrated in Fig. 3.

#### NSUNs crosstalk with transcriptional factors

Transcription factors regulate gene expression by binding to specific DNA sequences, thus influencing chromatin structure and transcriptional activity [67]. Research reveals that transcription factors can upregulate NSUN2 expression via direct interaction with its promoter [31, 68–71]. In esophageal squamous cell carcinoma (ESCC), Su et al. demonstrated that E2F Transcription Factor 1 (E2F1) bound to the NSUN2 promoter, driving its expression, thereby promoting ESCC cell proliferation [68]. In ovarian cancer, Liu et al. found that NSUN2 mediated m^5^C methylation in the 3′-UTR of *E2F1* mRNA, with YBX1 further stabilizing *E2F1* mRNA. E2F1 subsequently bound to the NSUN2 promoter, establishing a positive feedback loop that drives tumorigenesis and metastasis [69]. Additionally, in gastric cancer (GC) peritoneal metastasis, Liu et al. identified that peritoneal adipocytes provided fatty acids to GC cells, which upregulated E2F1, further promoting NSUN2 expression through cis-regulatory elements, promoting peritoneal metastasis and GC colonization [70]. In lung adenocarcinoma (LUAD), Du et al. found that MYC-associated zinc finger protein (MAZ) bound to the NSUN2 promoter, enhancing its transcription and NSUN2 expression to accelerated LUAD progression [71]. Likewise, in PCa, Zhu et al. demonstrated that the pre-mRNA of *androgen receptor (AR)* is modified by NSUN2 to maintain its stability, while AR acts as a transcription factor to upregulate NSUN2 expression. In this way, a positive feedback loop is formed between NSUN2 and AR to promote the progression of prostate cancer. More importantly, the AR inhibitor enzalutamide can reduce NSUN2 expression and decrease the level of m^5^C modification in prostate cancer cells. This suggests that inhibiting the expression of NSUNs at the transcription factor level is a feasible strategy for cancer treatment [31].

#### NSUNs regulated by NcRNAs

The expression of NSUNs in tumors is regulated by non-coding RNAs (ncRNAs), such as miRNAs and lncRNAs [72–78].

A large number of current studies have shown that lncRNAs can regulate the expression of NSUNs proteins. For example, lncRNA *hPVT1* upregulates NSUN1 by enhancing the stability of NSUN1 protein, thereby promoting the proliferation of HCC cells [72]. Similar to the aforementioned studies, the stability of NSUN2 is also regulated by long non-coding RNAs (lncRNAs), including lncRNA *HOST2, DIAPH2-AS1, LINC00618* and *FOXC2-AS* [73–76]. Specifically, in GC, Li et al. identified that the 385–815nt fragment of lncRNA *DIAPH2-AS1* bound to the 462–614aa region of NSUN2, masking the K577 and K579 ubiquitination sites and inhibiting ubiquitin-proteasome-mediated degradation to stabilize NSUN2, thus sustaining GC progression [73]. The 1-101nt region of *LINC00618* binds to NSUN2, inhibiting the ubiquitin-proteasome pathway-induced degradation of NSUN2 in HCC cells [75]. Unlike the aforementioned studies, lncRNA *HOST2* stabilizes *NSUN2* mRNA in an ELAVL1-dependent indirect manner, thereby maintaining NSUN2 protein expression. This finding further expands the expression regulation patterns of NSUNs mediated by lncRNAs [76]. These studies not only deepen the understanding of the mechanisms underlying the role of the lncRNA-NSUNs regulatory network in cancer, but also highlight the therapeutic potential of targeting lncRNAs upstream of NSUNs. By interfering with specific lncRNAs to restore the normal expression and function of NSUNs, it is expected to provide a breakthrough strategy for the development of novel epigenetic targeted therapies, and open up new research directions and clinical translation pathways for cancer treatment.

Furthermore, other ncRNAs can also mediate the abnormal expression of NSUNs in cancer. For example, in uveal melanoma (UM), Luo et al. demonstrated that *miR-124a* modulates NSUN2-mediated m^5^C modification by binding to 3’-untranslated region (3’-UTR) region of NSUN2. Downregulation of *miR-124a* leads in increased NSUN2 expression, which subsequently promotes UM cell proliferation and migration [77]. Furthermore, Qin et al. discovered that *piR-28846* can regulate NSUN2 expression. *piR-28846* binds to NSUN2 and downregulating its expression reduces the stability of the target *KPNA2* mRNA and the level of protein. At the same time, the downregulation of *KPNA2* level will irremediably down-regulate the expression of NSUN2, thereby inhibiting ovarian cancer cell growth [78]. These studies reveal that ncRNAs such as miRNAs and piRNAs precisely regulate the expression of NSUNs through multi-dimensional mechanisms including direct targeting or feedback loops, thereby affecting the epigenetic status and malignant biological behaviors of tumors.

Targeted intervention on these specific ncRNAs (e.g., mimic or antagomir strategies) is expected to precisely regulate the functional network of NSUNs, reshape the epigenetic status of cancer cells, and reverse their malignant phenotypes. This thus provides a novel paradigm and translational pathway for the development of RNA-based personalized cancer therapies.

#### PTMs positively regulate NSUNs

Post-translational modifications (PTMs) play a significant role in regulating gene expression in cancer cells [79], thereby influencing NSUNs stability and activity.

In terms of maintaining the stability of NSUNs, NSUNs are regulated by histone PTMs. A case in point, in endometrial cancer (EC), Chen et al. reported that reduced KDM5A expression resulted in elevated H3K4me3 levels, driving NSUN2 upregulation and enhancing EC cell proliferation [80]. More importantly, its protein stability is affected by its own PTMs. For instance, Hu et al. demonstrated that in GC, small ubiquitin-like modifier (SUMO)-2/3 interacted with the SIM (236–240aa) domain of NSUN2 through non-covalent bonds, stabilizing the protein and promoting its nuclear translocation, thereby supporting GC cell proliferation and metastasis [81]. Ubiquitin-specific peptidase 8 (USP8) stabilizes NSUN4 by inhibiting the specific K11-linked polyubiquitination of NSUN4 [82]. Meanwhile, as the primary function of NSUNs, their catalytic activity is also regulated by PTMs [83–85]. For instance, Chen et al. further found that in colorectal cancer (CRC), lactate accumulation, due to glycolysis and lactate production increased, On the one hand, promotes NSUN2 transcription through H3K18 lactylation, On the other hand, and lactate also induces NSUN2 lactylation at the Lys356 (K356) to enhance NSUN2 activity, creating a positive feedback loop that supports tumor growth and metastasis [83]. Meanwhile, Niu et al. discovered that NAA10 functions as a lactyltransferase, catalyzing lactylation at lysine 508 (K508) of NSUN2 upon lactate treatment. This PTM enhances NSUN2 activity, leading to increased m⁵C methylation. The NSUN2-mediated m⁵C modification subsequently promotes GCLC accumulation, thereby protecting gastric cancer cells from ferroptosis [84]. CDK13 interacts with the RNA methyltransferase NSUN5 and promotes the phosphorylation of NSUN5 at Ser327. The phosphorylated NSUN5 catalyzes the m^5^C modification of *ACC1* mRNA, thereby facilitating the progression of PCa [85].

Collectively, these studies demonstrate that dysregulation of NSUNs occurs through diverse mechanisms. In-depth exploration of the pathways underlying their aberrant expression represents a critical breakthrough opportunity for cancer therapy. Notably, PTM-mediated NSUN dysregulation offers particularly promising therapeutic potential. Elucidating key modification sites on NSUN2 and targeting these sites may enable novel drug development strategies, such as PROTACs or molecular glues, ultimately improving tumor prognosis.

### What downstream mechanisms are regulated by NSUNs

**Fig. 4:**
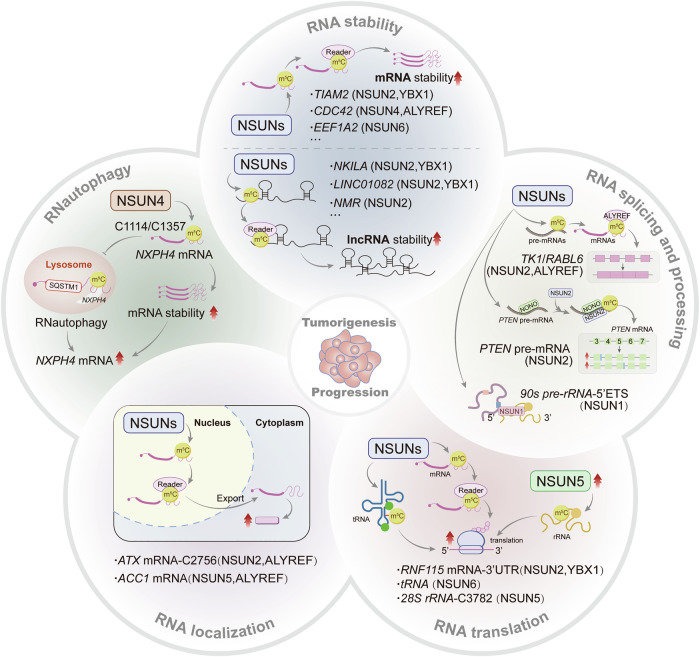
The downstream mechanism of NSUNs-mediated m^5^C modification in regulating RNA metabolism. NSUNs-mediated m^5^C modification influences tumor progression by regulating RNA metabolism. These mechanisms primarily regulate RNA stability, splicing and processing, translation, localization, and the novel mechanism of RNA autophagy [41, 45, 46, 58, 63, 85–107].

The previous section addressed the upstream mechanisms responsible for NSUN dysregulation. This section focused on the downstream effects of NSUN-mediated m^5^C modification in RNA metabolism, including its impact on RNA stability, splicing, processing, translation, localization, and the newly identified mechanism of RNA autophagy, all of which play a role in tumor progression (Fig. 4 and Table 2).

**Table 2:** The specific mechanism by which NSUNs regulate RNA metabolism.

Writer	Target RNA	m^5^C Site	Mechanism	References
NSUN1	*90* *s pre-*rRNA	5′ETS	RNA Splicing and Processing	[41]
NSUN2	*TIAM2*		RNA stability	[86]
	lncRNA *NKILA*		RNA stability	[91]
	*RNF115*	3’UTR	Translation Regulation	[101]
	*RABL6, TK1*	*RABL6* (5′UTR), *TK1* (3′UTR)	RNA Splicing and Processing	[97]
	*PTEN*		RNA Splicing and Processing	[99]
	*ATX*	C2756 (3′UTR)	RNA Localization	[105]
	*LINC01082*		RNA stability	[92]
	lncRNA *H19*	C986	RNA stability	[58]
	*LINC00324*	C1455	RNA stability	[93]
	lncRNA *NR_033928*	C154	RNA stability	[96]
	lncRNA *MALAT1*		RNA stability	[95]
	lncRNA *SNHG15*		RNA stability	[94]
	lncRNA *NMR*		Competitively inhibit the methylation of mRNA	[59]
NSUN4	*NXPH4*	C1114, C1357	RNautophagy	[107]
	*CDC42*	3′UTR	RNA stability	[87]
NSUN5	*ACC1*		RNA Localization	[85]
	*28S* rRNA	C3782	Translation Regulation	[102]
NSUN6	*tRNA*		Translation Regulation	[104]
	*EEF1A2*		RNA stability	[63]

#### NSUNs regulate mRNA stability

RNA stability is essential for understanding gene expression and biological processes. The m⁵C modification mediated by NSUN plays a significant role in regulating the stability of mRNA [63, 86–90]. For example, in pancreatic cancer (PC), Zhang et al. demonstrated that elevated NSUN2 levels catalyzed m^5^C methylation on *TIAM2*, stabilizing and upregulating its expression in a YBX1-dependent manner, thereby promoting tumorigenesis [86]. Furthermore, Zhao et al. showed that in glioma, NSUN4, in collaboration with ALYREF, catalyzes m^5^C methylation of *CDC42* mRNA, thereby stabilizing it and activating the PI3K-AKT pathway, which drives glioma progression [87]. Hu et al. found that NSUN6 enhances the stability of *EEF1A2* mRNA via m^5^C modification, thereby promoting the malignant progression of OS [63].

#### NSUNs regulate lncRNA stability

Long non-coding RNAs (lncRNAs) serve as key regulators of tumorigenesis and are subject to stability modulation by NSUNs [58, 91–96]. For example, Zheng et al. observed in cholangiocarcinoma (CCA) that NSUN2, in association with YBX1, stabilized lncRNA *NKILA* through m^5^C methylation, preventing its degradation. *NKILA*, in turn, regulated *miR-582-3p* expression via m^6^A modification, which increased YAP1 levels, thereby facilitating CCA proliferation and metastasis both in vitro and in vivo [91]. NSUN2-mediated m^5^C modification stabilizes *LINC01082* through interaction with the m^5^C reader protein YBX1, increasing *LINC01082* expression, which results in reduced proliferation and migration, as well as increased apoptosis of osteosarcoma (OS) cells [92]. In addition, a large body of studies has shown that the stability of lncRNAs can be regulated by NSUNs, including lncRNA *H19, LINC00324*, lncRNA *NR_033928, MALAT1*, and lncRNA *SNHG15* [58, 93–96]. Interestingly, Li et al. found that lncRNA *NMR* is methylated by NSUN2, which may competitively inhibit the methylation of mRNA [59]. Of greater importance, another studies demonstrated that NSUN2 catalyzes m^5^C modification of lncRNA *MALAT1* to stabilize *MALAT1*, thereby promoting its expression, leading to sorafenib resistance. More importantly, the combination of the MALAT1 inhibitor MALAT1-IN1 with sorafenib significantly enhanced the therapeutic efficacy of sorafenib in the treatment of HCC both in vitro and in vivo [95]. This finding highlights the therapeutic potential of targeting m^5^C-modified lncRNAs in reversing drug resistance. It also suggests that targeting the NSUN2-m^5^C-lncRNA axis, or developing small-molecule inhibitors or oligonucleotide-based drugs against m^5^C-modified lncRNAs, is expected to become a new strategy for overcoming tumor drug resistance and developing RNA modification-based combination therapy, which holds broad prospects for clinical translation.

#### NSUNs regulate RNA splicing and processing

In addition to modulating RNA stability, NSUN proteins influence RNA splicing and processing [41, 97–100]. In UCB, Wang et al. demonstrated that upregulation of NSUN2 enhanced m^5^C methylation at the *RABL6* (5′UTR) and *TK1* (3′UTR) regions, promoting mRNA splicing and stability through ALYREF, thus supporting UCB cell proliferation and migration [97]. Zhao et al. identified that NONO interacted directly with *PTEN* pre-mRNA and recruited the RNA m^5^C methyltransferase NSUN2 to modulate alternative splicing of *PTEN* introns, thereby promoting GC progression [99]. Furthermore, Liao et al. emphasized the critical function of NSUN1 in cell proliferation, rRNA processing, and ribosome biogenesis, noting that NSUN1 bound the 5′external transcribed spacer (5′ETS) region of pre-rRNA transcripts and regulated their processing by forming non-catalytic complexes with box C/D small nucleolar RNAs (snoRNAs) [41].

#### NSUNs regulate translation regulation

NSUNs are also involved in translation regulation [46, 101–104]. Li et al. revealed that NSUN2 catalyzes m⁵C modification in the 3’-UTR of *RNF115* mRNA. YBX1 recognizes m⁵C-modified sites in the *RNF115* 3’-UTR and interacts with *eIF4A1* to bridge the 5’-UTR region, facilitating mRNA circularization and translational activation—a mechanism driving HCC progression [101]. In glioblastoma (GBM), NSUN5 drove tumorigenesis by catalyzing m^5^C methylation at the C3782 site of *28S* rRNA, thereby enhancing protein translation [46]. Janin et al. further reported that in gliomas, depletion of NSUN5 resulted in the unmethylated C3782 position on *28S* rRNA, leading to a global reduction in protein synthesis and activating an adaptive translation program for cellular survival under stress [102]. In HCC, Zhang et al. indicated that NSUN5 enhanced ribosome function and protein translation, potentially promoting HCC cell proliferation and migration [103]. In ESCC, Han et al. showed that reduced NSUN6 expression accelerated ESCC cell proliferation, migration, and invasion. Mechanistically, NSUN6-mediated m^5^C modification of specific tRNA selectively improves the translational efficiency of *CDH1* mRNA in a codon-dependent manner, thereby influencing ESCC progression through modulation of E-cadherin expression [104].

#### NSUNs regulate RNA localization

Emerging studies suggest that NSUNs also play a role in RNA localization within cells, contributing to tumorigenesis [45, 85, 105]. Yang et al. first reported that NSUN2-mediated m^5^C methylation regulated mRNA export via ALYREF [45]. Subsequently, Xu et al. demonstrated in glioma cells that NSUN2 catalyzed m^5^C methylation at position 2756 in the 3’-UTR of *ATX* mRNA, promoting its export from the nucleus to the cytoplasm via ALYREF, thus enhancing cancer cell migration [105]. Similarly, Zhang et al. showed that phosphorylated NSUN5 catalyzed m^5^C modification of *ACC1* mRNA. ALYREF bound to the m^5^C-modified *ACC1* mRNA, stabilizing it and facilitating its nuclear export, which drives *ACC1* expression and accelerates tumor progression in PCa cells [85].

#### NSUNs regulate RNautophagy

RNautophagy is a recently characterized form of autophagy, and it is responsible for RNA degradation within autophagosomes [106]. This form of autophagy (RNautophagy) has been implicated in NSUN-mediated modifications. Yang et al. demonstrated that in CRC, upregulated NSUN4 catalyzed m^5^C methylation at positions 1114 and 1357 of *NXPH4*. This modification disrupts the interaction between *NXPH4* mRNA and SQSTM1, thus preventing its degradation via RNautophagy. Simultaneously, the modification stabilizes *NXPH4* mRNA, inhibits PHD4 binding to HIF1A, prevents the degradation, and enhances HIF signaling, thereby promoting CRC cell proliferation [107].

These observations suggest that NSUN proteins regulate RNA abundance by catalyzing m^5^C modifications, which affect RNA stability, splicing, processing, translation, and localization, leading to the dysregulation of target proteins that contribute to tumorigenesis. Targeting the expression or activity of NSUNs could counteract m^5^C-modification-driven tumorigenesis, offering potential therapeutic strategies for cancer treatment.

### What biological functions are affected by NSUNs in tumors

**Fig. 5:**
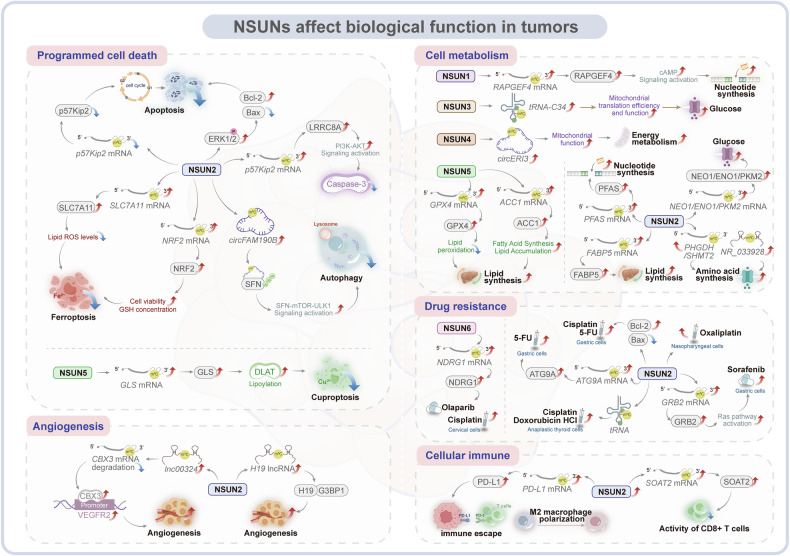
The biological functions of NSUNs in tumors. NSUNs regulate tumor functions and thereby influence tumor progression. They participate in the regulation of programmed cell death, cellular immune, angiogenesis, durg resistance, and metabolic reprogramming in tumor cells, including glucose metabolism, nucleotide metabolism, amino acid metabolism, lipid metabolism and energy metabolism [16, 30, 58, 66, 80, 83, 85, 89, 93, 96, 108–138].

NSUN proteins are widely expressed in various tissues and are strongly associated with tumorigenesis and cancer progression. These proteins regulate key cellular processes, including programmed cell death, cell cycle progression, angiogenesis, metabolic reprogramming, tumor immunity, and drug resistance in tumor cells (Fig. 5).

#### NSUNs affect programmed cell death

Apoptosis, a key mechanism of programmed cell death, is critical for maintaining homeostasis, development, and immune function [108]. The cell cycle modulates apoptosis, with disruptions in its dynamics influencing the initiation and progression of apoptotic events [109]. Numerous studies have shown that NSUN proteins inhibit cell cycle arrest and reduce apoptosis in cancer [89, 110, 111]. For instance, Shen et al. demonstrated that elevated NSUN2 expression in GC suppressed apoptosis and promotes cell proliferation. Specifically, NSUN2 activates *ERK1/2* signaling via m^5^C methylation, preventing G1/S checkpoint arrest. Additionally, NSUN2 upregulates the anti-apoptotic protein Bcl-2 and downregulates the pro-apoptotic protein Bax, effectively inhibiting apoptosis in GC cells [110]. NSUN2 also promotes GC progression through an alternative mechanism by inhibiting m^5^C methylation in the 3′-UTR of *p57*^*Kip2*^ to reduces mRNA stability and expression, thereby preventing G1/G0 arrest and promoting tumor progression [89]. In CC, NSUN2 catalyzes m^5^C methylation of *LRRC8A* mRNA, stabilizing and enhancing its expression. This upregulation activates the PI3K-AKT pathway and inhibits Caspase-3, a key apoptosis-regulating protein, thereby contributing to tumor growth [111].

Ferroptosis, a recently recognized form of programmed cell death, results from the accumulation of iron-dependent lipid peroxides [112]. Evidence suggests that NSUN proteins, beyond their role in apoptosis, are intricately involved in the regulation of ferroptosis. In NSCLC, for example, Chen et al. reported that NSUN2 expression was elevated and, in cooperation with YBX1, targets the 5′UTR of *NRF2* mRNA, promoting its stability through m^5^C methylation. This modification diminishes the sensitivity of NSCLC cells to ferroptosis inducers, thereby enhancing ferroptosis resistance and supporting tumor cell proliferation, migration, and invasion [113]. Similarly, in EC, Chen et al. demonstrated that NSUN2, together with YBX1, stabilized *SLC7A11* mRNA, a ferroptosis inhibitor, through m^5^C methylation. This modification increases lipid peroxidation, bolstering EC cells’ resistance to ferroptosis and promoting tumor progression [80].

Beyond ferroptosis regulation, NSUN family proteins also mediate tumor cell cuproptosis. For instance, Shu et al. demonstrated that NSUN5 facilitates glutaminase (GLS) accumulation through m⁵C modification at position C137 in the *GLS* mRNA UTR region. This post-transcriptional regulation enhances GLS-mediated copper chelation, thereby mitigating cuproptosis and promoting cholangiocarcinoma (CCA) progression [114].

Autophagy is a form of programmed cell death [115]. NSUN2 mediates m⁵C modification of *circFAM190B* and enhances its stability in an m⁵C-dependent manner. Mechanistically, *circFAM190B* directly targets stratifin (SFN) and modulates its ubiquitination, thereby suppressing cellular autophagy through the SFN/mTOR/ULK1 signaling axis. This regulatory cascade ultimately promotes lung cancer progression [116].

These studies highlight the critical role of programmed cell death in tumorigenesis, suggesting that targeting NSUNs to enhance tumor cell death and impair proliferation could provide novel therapeutic avenues for cancer treatment.

#### NSUNs affect angiogenesis

Tumor angiogenesis plays a critical role in oncogenesis, progression, and metastasis, positioning it as a key therapeutic target in cancer treatment [117]. Research indicates that NSUN2 is closely involved in regulating angiogenesis in cancer cells. For example, Pan et al. demonstrated that in Glioblastoma endothelial cells (GECs), elevated NSUN2 levels catalyzed m^5^C methylation at the C1455 site of *LINC00324*, stabilizing this lncRNA. Stabilized *LINC00324* competes with *CBX3* mRNA for binding to AUH protein, reducing *CBX3* mRNA degradation. As a result, CBX3 binds to the VEGFR2 promoter, enhancing vascular endothelial growth factor receptor 2 (VEGFR2) expression and promoting angiogenesis, cell proliferation, and migration in GECs [93]. In HCC, Sun et al. showed that high NSUN2 expression targeted the C986 site of *H19* through m^5^C methylation, increasing the stability of this lncRNA. Methylated *H19* interacts with the oncogene G3BP1, promoting angiogenesis and supporting HCC cell proliferation, migration, and invasion [58].

Angiogenesis is the key process in the growth, survival, invasiveness, and metastasis of cancerous tumors, so antiangiogenic chemotherapy is a novel approach to the treatment of drug resistance [118]. These results suggest that modulating RNA m^5^C modifications to influence anti-angiogenic factors could offer a promising strategy for cancer therapy.

#### NSUNs affect cellular metabolism

Cancer metabolism, a central hallmark of malignancy, is critical for tumor initiation and progression [119]. NSUN proteins regulate key metabolic processes, including glucose, lipid, amino acid metabolism and energy metabolism, thereby influencing cancer development [16, 66, 83, 85, 96, 120–127].

Glucose metabolism, essential for tumor progression, is modulated by NSUNs in various cancers [83, 121, 122]. In CRC, Chen et al. demonstrated that lactate-induced *H3K18* lactylation activated NSUN2 transcription, which in turn triggers NSUN2 lactylation at Lys356 (K356), enhancing its RNA-binding affinity. In collaboration with YBX1, NSUN2 stabilizes *ENO1* mRNA through m^5^C methylation, driving glucose metabolic reprogramming [83]. Furthermore, NSUN2 enhances m⁵C methylation at the C773 site within the 3’-UTR of *PKM2* mRNA, thereby stabilizing *PKM2* transcripts. This upregulation of *PKM2* expression promotes glycolytic flux and drives HCC progression [121]. Wang et al. demonstrated that NSUN2 promotes glycolysis in renal cancer by maintaining the stability of *NEO1* mRNA [122].

NSUNs also regulate lipid metabolism in cancer [85, 123, 124]. In COAD, Chen et al. demonstrated that NSUN5, in collaboration with RBM15B and IGFBP2, stabilizes *GPX4* mRNA through m^5^C or m^6^A modifications, reducing lipid peroxidation and activating the cGAS-STING pathway to promote anti-tumor immune responses [123]. In OS, Yang et al. found that elevated NSUN2 expression targeted *FABP5* mRNA at C271 and C321, stabilizing it via m^5^C modification. This modification promotes neutral lipid lipolysis and fatty acid metabolism, thereby enhancing OS cell proliferation, invasion, and migration [124]. Additionally, Zhang et al. showed that phosphorylated NSUN5 catalyzed the m^5^C modification of *ACC1* mRNA, after which ALYREF bound to the m^5^C-modified *ACC1* mRNA, improving its stability and promoting nuclear export. This process increases ACC1 expression and lipid deposition in PCa cells, supporting tumor progression [85].

Amino acid metabolism, integral to tumor biology, is closely associated with NSUN proteins. Li et al. demonstrated that in acute myeloid leukemia (AML), NSUN2 upregulates the expression of two key enzymes in serine metabolism, PHGDH and SHMT2, thereby affecting serine and one-carbon metabolism to sustain the proliferation of AML cells [125]. Similarly, Fang et al. reported high NSUN2 expression in GC, where it catalyzed m^5^C methylation of lncRNA *NR033928*, stabilizing it. *NR_033928* subsequently interacts with the IGF2BP3/HUR complex to stabilize *GLS* mRNA, increasing GLS expression, which regulates glutamine metabolism and enhances GC cell proliferation while inhibiting apoptosis [96].

Beyond amino acid metabolism, NSUNs also regulate nucleotide metabolism, including purine synthesis and cAMP signaling. In retinoblastoma (RB), Zuo et al. demonstrated that elevated NSUN2 expression stabilized *PFAS* mRNA via m^5^C methylation. *PFAS* promotes purine synthesis by increasing intermediate metabolites (IMP, AMP, and GMP), thereby driving RB cell proliferation [66]. In high-grade serous ovarian cancer (HGSOC), Yang et al. showed that NSUN1 overexpression modulated *RAPGEF4* mRNA through m^5^C methylation, influencing the cAMP signaling pathway and supporting HGSOC cell survival in vitro [126].

Concurrently, NSUNs play pivotal roles in mitochondrial energy metabolism. Wu et al. demonstrated that NSUN4-mediated m⁵C modification enhances *circERI3* stability. Mechanistically, *circERI3* binds to and regulates the ubiquitination of DNA damage-binding protein 1 (DDB1), increasing its protein stability. This DDB1 accumulation subsequently promotes peroxisome proliferator-activated receptor gamma coactivator 1-alpha (PGC-1α) transcription, thereby modulating mitochondrial function and energy metabolism to drive lung cancer progression [127]. Similarly, Delaunay et al. reported that oral cancer cells with reduced mitochondrial m^5^C levels showed decreased oxidative phosphorylation (OXPHOS) and reliance on glycolysis for energy. Elevated NSUN3 expression catalyzes m^5^C modification at mitochondrial *tRNA-C34*, increasing m^5^C and f^5^C levels, which enhances mitochondrial translation, supports OXPHOS, and promotes cancer cell invasion and metastasis from the primary tumor [16].

These results illustrate how NSUNs alter cancer cell metabolism through m^5^C methylation. Targeting NSUNs may disrupt tumor cell metabolic states, presenting potential strategies to inhibit tumor growth and improve cancer treatment outcomes.

#### NSUNs affect drug resistance

**Fig. 6:**
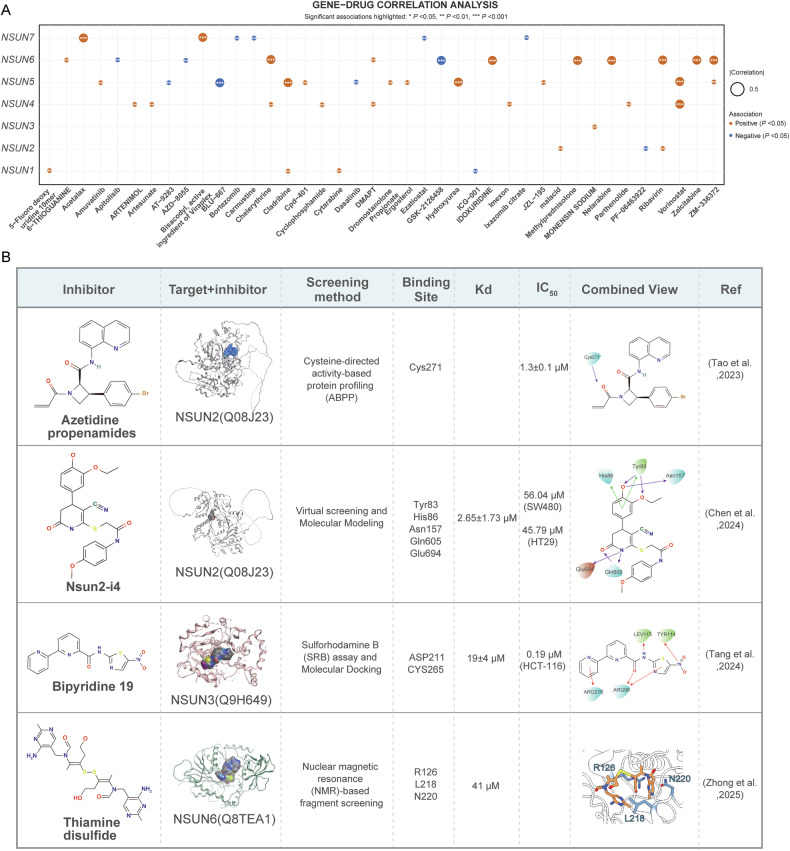
NSUNs as potential targets for tumor therapy. **A** The correlation analysis between NSUNs gene expression data from the CellMiner database (https://discover.nci.nih.gov/cellminer/home.do) and drug sensitivity information from the Genomics of Drug Sensitivity in Cancer (GDSC, https://www.cancerrxgene.org/) revealed a significant association between NSUNs expression and response to various anticancer agents (*R* > 0.36). **B** Core Characteristics of NSUNs-Targeted Inhibitors. Drawing on existing research on NSUNs inhibitors, this figure presents key information on current NSUNs inhibitors, including their chemical structures, screening methods, binding sites and modes, dissociation constants (Kd), half-maximal inhibitory concentrations (IC₅₀), and relevant research references. The inhibitors including Bipyridine 19 [140], NSUN2-i4(NSUNi) [83, 100], Azetidine propenamides [139] and thiamine disulfide [141].

Cancer drug resistance remains a significant challenge in tumor progression [128]. Aberrant NSUN expression can regulate cancer cell sensitivity to anti-cancer therapies, thereby contributing to drug resistance regulation. NSUNs influence resistance to conventional chemotherapies, including cisplatin, oxaliplatin, doxorubicin, and others, through mediation of RNA m^5^C modifications [110, 129–135]. For instance, Shen et al. demonstrated that NSUN2 enhanced GC cell sensitivity to chemotherapeutic agents like cisplatin (CDDP) and 5-Fluorouracil (5-FU) [110]. And Huang et al. discovered that YBX1 mediates the m^5^C modification of *ATG9A* mRNA through NSUN2, thereby enhancing autophagic activity and conferring resistance to 5-FU [132]. Li et al. found that NSUN2 knockout diminished the tolerance of ATC cells to low-dose cisplatin or doxorubicin hydrochloride [133]. Tong et al. reported that in NPC, cells with elevated NSUN2 levels exhibited increased resistance to oxaliplatin [30]. Additionally, NSUNs mediate resistance to targeted therapies such as sorafenib, osimertinib, and olaparib [134, 135]. Research by Song et al. revealed that in liver cancer, sorafenib—a Raf phosphorylation inhibitor targeting Ras activity—was commonly used in HCC treatment. NSUN2 inhibits Ras activation and reduces p-Erk levels, enhancing HCC cell sensitivity to sorafenib [134]. Similarly, Yu et al. demonstrated that high NSUN6 expression can decrease CC cell sensitivity to cisplatin and olaparib and promote the radioresistance [135]. Moreover, based on the NSUNs gene expression matrix from the CellMiner database (https://discover.nci.nih.gov/cellminer/home.do) and the drug sensitivity data from the Genomics of Drug Sensitivity in Cancer (GDSC, https://www.cancerrxgene.org/), we analyzed the correlation between NSUN expression and drug sensitivity. This analysis revealed a positive correlation between NSUN expression and the sensitivity to certain oncological drugs (Fig. 6A (*R* > 0.36) and Table 3), suggesting that NSUN proteins are promising targets for overcoming drug resistance and improving patient outcomes.

**Table 3:** The correlation analysis between NSUNs gene expression data and drug sensitivity information.

Gene	Drug	Correlation	*P* value	Gene	Drug	Correlation	*P* value
NSUN1	Cladribine	0.4075	0.0012	NSUN5	Thiotepa	0.3192	0.0129
NSUN1	5-Fluoro deoxy uridine 10mer	0.3787	0.0028	NSUN5	IDOXURIDINE	0.3181	0.0132
NSUN1	Cytarabine	0.3637	0.0043	NSUN5	Clofarabine	0.3162	0.0138
NSUN1	Cisplatin	0.3371	0.0084	NSUN5	Cytarabine	0.3124	0.0151
NSUN1	Gemcitabine	0.3324	0.0095	NSUN5	Gemcitabine	0.3090	0.0163
NSUN1	Clofarabine	0.3265	0.0109	NSUN5	Mocetinostat	0.3035	0.0184
NSUN1	LY-2606368	0.3213	0.0123	NSUN5	Fludarabine	0.3025	0.0188
NSUN1	Cpd-401	0.3204	0.0126	NSUN5	Belinostat	0.2972	0.0211
NSUN1	Carboplatin	0.3132	0.0148	NSUN5	ciclosporin	0.2952	0.0220
NSUN1	Raltitrexed	0.3100	0.0159	NSUN5	Karenitecin	0.2930	0.0231
NSUN1	Fludarabine	0.2989	0.0204	NSUN5	MONENSIN SODIUM	0.2912	0.0240
NSUN1	Thiotepa	0.2963	0.0215	NSUN5	CAMPTOTHECIN	0.2847	0.0275
NSUN1	Ancitabine hydrochloride	0.2939	0.0226	NSUN5	Pralatrexate	0.2825	0.0287
NSUN1	Enzalutamide	0.2918	0.0237	NSUN5	Pracinostat	0.2808	0.0298
NSUN1	TRIMETREXATE GLUCURONATE	0.2891	0.0251	NSUN5	Asparaginase	0.2749	0.0335
NSUN1	Nelarabine	0.2888	0.0252	NSUN5	Nitrogen mustard	0.2719	0.0356
NSUN1	METHOTREXATE	0.2887	0.0253	NSUN5	Fulvestrant	0.2714	0.0360
NSUN1	Karenitecin	0.2873	0.0260	NSUN5	Carboplatin	0.2705	0.0366
NSUN1	malacid	0.2871	0.0261	NSUN5	LMP776	0.2692	0.0376
NSUN1	Triapine	0.2812	0.0296	NSUN5	8-Chloro-adenosine	0.2676	0.0387
NSUN1	Methylprednisolone	0.2809	0.0297	NSUN5	XL-147	0.2635	0.0419
NSUN1	Zalcitabine	0.2775	0.0318	NSUN5	Tipifarnib	0.2625	0.0427
NSUN1	ANCITABINE HYDROCHLORIDE	0.2727	0.0350	NSUN5	Ancitabine hydrochloride	0.2584	0.0462
NSUN1	VE-821	0.2688	0.0379	NSUN5	tfdu	0.2582	0.0464
NSUN1	Pemetrexed	0.2657	0.0402	NSUN5	DMAPT	0.2564	0.0480
NSUN1	Triethylenemelamine	0.2626	0.0426	NSUN5	5-Fluoro deoxy uridine 10mer	0.2559	0.0484
NSUN1	Chlorambucil	0.2609	0.0440	NSUN5	Imexon	0.2552	0.0491
NSUN1	DIGOXIN	0.2596	0.0452	NSUN5	Dovitinib	−0.2547	0.0495
NSUN1	Floxuridine	0.2579	0.0467	NSUN5	Rebimastat	−0.3075	0.0169
NSUN1	Asparaginase	0.2553	0.0489	NSUN5	Pluripotin	−0.3104	0.0158
NSUN1	Pipobroman	0.2546	0.0496	NSUN5	JNJ−42756493	−0.3210	0.0124
NSUN1	EMD-534085	−0.2644	0.0412	NSUN5	Staurosporine	−0.3370	0.0085
NSUN1	Paclitaxel	−0.2727	0.0350	NSUN5	AT-9283	−0.3825	0.0026
NSUN1	VINORELBINE	−0.2782	0.0314	NSUN5	Dasatinib	−0.3912	0.0020
NSUN1	Vinorelbine	−0.2828	0.0285	NSUN5	BLU-667	−0.4308	0.0006
NSUN1	Kahalide F	−0.2991	0.0203	NSUN6	Nelarabine	0.5371	0.0000
NSUN1	TAK-901	−0.3015	0.0192	NSUN6	Methylprednisolone	0.4901	0.0000
NSUN1	PKI-587	−0.3135	0.0147	NSUN6	Ribavirin	0.4808	0.0001
NSUN1	ICG-001	−0.3623	0.0044	NSUN6	Zalcitabine	0.4725	0.0001
NSUN2	malacid	0.3846	0.0024	NSUN6	IDOXURIDINE	0.4493	0.0003
NSUN2	Ribavirin	0.3714	0.0035	NSUN6	Chelerythrine	0.4374	0.0005
NSUN2	Methylprednisolone	0.3573	0.0051	NSUN6	ZM-336372	0.4163	0.0009
NSUN2	Econazole Nitrate	0.3092	0.0162	NSUN6	DMAPT	0.4081	0.0012
NSUN2	Dexamethasone Decadron	0.3028	0.0187	NSUN6	6-THIOGUANINE	0.3704	0.0036
NSUN2	Clofarabine	0.3026	0.0188	NSUN6	Crizotinib	0.3585	0.0049
NSUN2	ZM-336372	0.2953	0.0220	NSUN6	ST-3595	0.3531	0.0056
NSUN2	Allopurinol	0.2926	0.0233	NSUN6	PX-316	0.3523	0.0058
NSUN2	Fludarabine	0.2887	0.0253	NSUN6	6-Thioguanine	0.3479	0.0064
NSUN2	Pyrazoloacridine	0.2653	0.0405	NSUN6	Palbociclib	0.3457	0.0068
NSUN2	Cladribine	0.2571	0.0474	NSUN6	Ergosterol	0.3452	0.0069
NSUN2	Ruxolitinib	−0.2581	0.0465	NSUN6	Econazole nitrate	0.3416	0.0076
NSUN2	Nelfinavir	−0.2733	0.0346	NSUN6	PYRAZOLOACRIDINE	0.3405	0.0078
NSUN2	Denileukin Diftitox Ontak	−0.3050	0.0178	NSUN6	3-Bromopyruvate (acid)	0.3366	0.0085
NSUN2	Dovitinib	−0.3194	0.0129	NSUN6	8-Chloro-adenosine	0.3354	0.0088
NSUN2	Alectinib	−0.3239	0.0116	NSUN6	Cyclophosphamide	0.3332	0.0093
NSUN2	NMS-E628	−0.3567	0.0051	NSUN6	Pyrazoloacridine	0.3301	0.0100
NSUN2	PF-06463922	−0.3691	0.0037	NSUN6	Obatoclax	0.3208	0.0125
NSUN3	MONENSIN SODIUM	0.3637	0.0043	NSUN6	FENRETINIDE	0.3184	0.0132
NSUN3	ZM-336372	0.3588	0.0049	NSUN6	Navitoclax	0.3103	0.0158
NSUN3	Methylprednisolone	0.3372	0.0084	NSUN6	Imexon	0.3101	0.0159
NSUN3	SB-590885	0.3041	0.0182	NSUN6	ABL-001	0.3035	0.0184
NSUN3	Vorinostat	0.3029	0.0186	NSUN6	Fostamatinib	0.2981	0.0207
NSUN3	Econazole Nitrate	0.3006	0.0196	NSUN6	Ponatinib	0.2961	0.0216
NSUN3	Haloperidol	0.2999	0.0199	NSUN6	Dexrazoxane	0.2932	0.0230
NSUN3	Pracinostat	0.2959	0.0217	NSUN6	Oxaliplatin	0.2916	0.0238
NSUN3	AT-13387	0.2949	0.0222	NSUN6	AMG-900	0.2899	0.0246
NSUN3	LEE-011	0.2786	0.0311	NSUN6	Nilotinib	0.2899	0.0246
NSUN3	GW-5074	0.2766	0.0324	NSUN6	DACARBAZINE	0.2868	0.0263
NSUN3	SNS-314	0.2754	0.0332	NSUN6	Asparaginase	0.2857	0.0269
NSUN3	Nelarabine	0.2680	0.0384	NSUN6	AMONAFIDE	0.2854	0.0271
NSUN3	Abexinostat	0.2673	0.0389	NSUN6	Vorinostat	0.2846	0.0275
NSUN3	Fenretinide	0.2571	0.0474	NSUN6	ABT-737	0.2828	0.0286
NSUN3	ANCITABINE HYDROCHLORIDE	0.2564	0.0480	NSUN6	Ifosfamide	0.2806	0.0299
NSUN3	Zoledronate	−0.2574	0.0471	NSUN6	Dexamethasone Decadron	0.2765	0.0324
NSUN3	Dasatinib	−0.2838	0.0280	NSUN6	BP-1-102	0.2746	0.0337
NSUN3	Erlotinib	−0.2841	0.0278	NSUN6	malacid	0.2738	0.0343
NSUN3	JNJ-42756493	−0.3085	0.0165	NSUN6	Fluphenazine	0.2724	0.0352
NSUN4	Vorinostat	0.4253	0.0007	NSUN6	Tivozanib	0.2710	0.0362
NSUN4	Imexon	0.4036	0.0014	NSUN6	Imatinib	0.2704	0.0366
NSUN4	Parthenolide	0.3994	0.0016	NSUN6	TAE-684	0.2653	0.0405
NSUN4	ARTENIMOL	0.3846	0.0024	NSUN6	BX-912	0.2641	0.0415
NSUN4	DMAPT	0.3725	0.0034	NSUN6	Volasertib	0.2629	0.0424
NSUN4	Cyclophosphamide	0.3689	0.0037	NSUN6	Barasertib	0.2620	0.0431
NSUN4	Artesunate	0.3675	0.0039	NSUN6	CUDC-305	0.2578	0.0468
NSUN4	Chelerythrine	0.3675	0.0039	NSUN6	1,9-Pyrazoleanthrone	0.2576	0.0469
NSUN4	Mocetinostat	0.3526	0.0057	NSUN6	Fludarabine	0.2570	0.0474
NSUN4	8-Chloro-adenosine	0.3523	0.0058	NSUN6	pentamidine isethionate	−0.2666	0.0395
NSUN4	Masoprocol	0.3406	0.0078	NSUN6	Irofulven	−0.2793	0.0307
NSUN4	Artemether	0.3367	0.0085	NSUN6	Copanlisib	−0.2899	0.0247
NSUN4	Cladribine	0.3331	0.0093	NSUN6	Deforolimius	−0.2952	0.0220
NSUN4	Cpd-401	0.3216	0.0122	NSUN6	CH-5132799	−0.3076	0.0168
NSUN4	3-Bromopyruvate (acid)	0.3170	0.0136	NSUN6	PF-04691502	−0.3116	0.0154
NSUN4	Megestrol acetate	0.3073	0.0169	NSUN6	Everolimus	−0.3493	0.0062
NSUN4	Carmustine	0.3023	0.0189	NSUN6	Pictilisib	−0.3538	0.0056
NSUN4	auranofin	0.2999	0.0199	NSUN6	AZD-8055	−0.3655	0.0041
NSUN4	Methylprednisolone	0.2983	0.0206	NSUN6	Apitolisib	−0.4068	0.0013
NSUN4	DACARBAZINE	0.2950	0.0221	NSUN6	GSK-2126458	−0.5280	0.0000
NSUN4	Nelarabine	0.2904	0.0244	NSUN7	Acetalax	0.4537	0.0003
NSUN4	HYPOTHEMYCIN	0.2902	0.0245	NSUN7	Bisacodyl, active ingredient of Viraplex	0.4469	0.0003
NSUN4	Amuvatinib	0.2859	0.0268	NSUN7	LOXO-101	0.3529	0.0057
NSUN4	Lomustine	0.2772	0.0320	NSUN7	Linsitinib	0.2614	0.0437
NSUN4	Fluphenazine	0.2665	0.0396	NSUN7	SR16157	0.2583	0.0463
NSUN4	Fostamatinib	0.2617	0.0434	NSUN7	Ribavirin	0.2548	0.0494
NSUN4	6-THIOGUANINE	0.2612	0.0438	NSUN7	Etoposide	−0.2558	0.0486
NSUN4	Fludarabine	0.2583	0.0463	NSUN7	Carboplatin	−0.2583	0.0463
NSUN4	Asparaginase	0.2583	0.0463	NSUN7	Pazopanib	−0.2618	0.0433
NSUN4	Econazole nitrate	0.2548	0.0494	NSUN7	BP-1-102	−0.2622	0.0430
NSUN4	ICG-001	−0.2668	0.0393	NSUN7	Cisplatin	−0.2637	0.0418
NSUN4	Dasatinib	−0.2906	0.0243	NSUN7	pralatrexate	−0.2639	0.0416
NSUN4	PKI-587	−0.3026	0.0188	NSUN7	Teniposide	−0.2670	0.0392
NSUN4	Kahalide F	−0.3564	0.0052	NSUN7	Lomustine	−0.2681	0.0384
NSUN5	Vorinostat	0.5133	0.0000	NSUN7	MITOXANTRONE	−0.2684	0.0381
NSUN5	Hydroxyurea	0.4642	0.0002	NSUN7	ARSENIC TRIOXIDE	−0.2689	0.0377
NSUN5	Cladribine	0.4221	0.0008	NSUN7	Estramustine	−0.2748	0.0336
NSUN5	Amuvatinib	0.3995	0.0016	NSUN7	Midostaurin	−0.2810	0.0297
NSUN5	Ergosterol	0.3926	0.0019	NSUN7	ONX-0914	−0.2812	0.0295
NSUN5	Dromostanolone Propionate	0.3862	0.0023	NSUN7	Depsipeptide	−0.2828	0.0286
NSUN5	Cpd-401	0.3765	0.0030	NSUN7	Pipamperone	−0.2856	0.0270
NSUN5	ZM-336372	0.3759	0.0031	NSUN7	Encorafenib	−0.2925	0.0233
NSUN5	JZL-195	0.3612	0.0046	NSUN7	Vincristine	−0.2973	0.0211
NSUN5	Pipobroman	0.3589	0.0049	NSUN7	DACARBAZINE	−0.3008	0.0195
NSUN5	Artemether	0.3588	0.0049	NSUN7	BLU-285	−0.3044	0.0181
NSUN5	Entinostat	0.3587	0.0049	NSUN7	Epirubicin	−0.3086	0.0164
NSUN5	Cyclophosphamide	0.3537	0.0056	NSUN7	auranofin	−0.3136	0.0147
NSUN5	LMP-400	0.3412	0.0076	NSUN7	Homoharringtonine	−0.3158	0.0140
NSUN5	Uracil mustard	0.3371	0.0084	NSUN7	Doxorubicin	−0.3210	0.0124
NSUN5	Abexinostat	0.3367	0.0085	NSUN7	COLCHICINE	−0.3228	0.0119
NSUN5	Chlorambucil	0.3340	0.0091	NSUN7	BN-2629	−0.3302	0.0100
NSUN5	Enzalutamide	0.3335	0.0092	NSUN7	Voreloxin	−0.3339	0.0091
NSUN5	Triethylenemelamine	0.3316	0.0097	NSUN7	ABT-199	−0.3401	0.0078
NSUN5	Nelarabine	0.3309	0.0098	NSUN7	Ixazomib	−0.3422	0.0074
NSUN5	Methylprednisolone	0.3293	0.0102	NSUN7	Arsenic trioxide	−0.3461	0.0068
NSUN5	Masoprocol	0.3283	0.0104	NSUN7	RAF-265	−0.3551	0.0054
NSUN5	TESTOLACTONE	0.3243	0.0115	NSUN7	Okadaic acid	−0.3598	0.0047
NSUN5	Iniparib	0.3241	0.0115	NSUN7	Ezatiostat	−0.3768	0.0030
NSUN5	Megestrol acetate	0.3241	0.0115	NSUN7	Bortezomib	−0.3796	0.0028
NSUN5	Chelerythrine	0.3228	0.0119	NSUN7	Ixazomib citrate	−0.3807	0.0027
NSUN5	Zalcitabine	0.3213	0.0123	NSUN7	Carmustine	−0.3960	0.0017

#### NSUNs affect cellular immune

Tumor immunity represents a complex biological process that plays pivotal roles in oncogenesis, cancer progression, and therapeutic responses. Emerging evidence demonstrates the crucial involvement of NSUN proteins in tumor immune regulation. For instance, Yang et al. revealed that the NSUN2-ALYREF complex stabilizes *PD-L1* mRNA in an m⁵C-dependent manner, thereby enhancing PD-L1 expression and facilitating tumor immune evasion [136]; Furthermore, in diffuse large B-cell lymphoma (DLBCL), tumor-derived exosomes mediate NSUN2 transfer between malignant cells, where NSUN2 maintains *PD-L1* mRNA stability through m⁵C modification, ultimately promoting DLBCL proliferation, M2 macrophage polarization, and immune escape [137]. Moreover, Jiang et al. demonstrated that NSUN2 promotes the m^5^C modification of *SOAT2*, enhances the reprogramming of energy metabolism, suppresses the activity and cytotoxicity of CD8^+^ T cells, and contributes to immune evasion [138]. These findings highlight the importance of elucidating NSUN-mediated immune regulatory mechanisms for developing more effective immunotherapies and improving clinical outcomes.

### Are NSUNs potential targets for tumor therapy?

This section reviews studies identifying NSUNs as key targets in tumor drug resistance and drug development, highlighting their potential roles in overcoming drug resistance and in the design of novel therapeutic agents.

#### NSUNs as targets for drug development

NSUN proteins not only contribute to cancer progression but also influence tumor sensitivity to antitumor therapies. Evidence suggests that targeting NSUNs could offer a novel therapeutic approach in cancer treatment. This section reviews the development of small molecule inhibitors targeting NSUN2. For example, Tao et al. used cysteine-directed activity-based protein profiling (ABPP) to identify stereoselective covalent inhibitors of NSUN2, specifically azetidine propenamides. These inhibitors were shown to block the catalytic activity at the C271 site in recombinant NSUN2 and disrupt the NSUN2-tRNA interaction in cancer cells, resulting in decreased tRNA m^5^C content and suppression of cancer progression [139]. Therefore, the development of NSUN inhibitors that selectively target the conserved catalytic cysteine via covalent chemistry could offer promising directions for anticancer drug discovery. Tang et al. designed and synthesized 90 novel bipyridine derivatives based on the structure of caerulomycin A, ultimately selecting B19 as a potent inhibitor of NSUN3. B19 binds specifically to NSUN3, activates the AMPK/STAR3 signaling pathway, inhibits STAR3 phosphorylation, disrupts mitochondrial energy metabolism, and suppresses tumor proliferation [140]. Meanwhile, thiamine disulfide, a non-SAM analog inhibitor of NSUN6, has recently been identified [141].

More importantly, an increasing number of targeted inhibitors have been developed for immunotherapy and chemoresistance sensitization. In a study, Chen et al. designed the small-molecule inhibitor Nsun2-i4, targeting the NSUN2 catalytic pocket. This inhibitor was found to interact with multiple NSUN2 residues, including Tyr83, His86, Asn157, Gln605, and Glu694, to hinder CRC progression. The safety, toxicity, and applicability of Nsun2-i4 were also assessed. Furthermore, a potential synergistic effect between Nsun2-i4 and PD-1 was observed, with the combination showing a significantly enhanced inhibitory effect on tumor growth compared to individual treatments [83]. In another study, Hou et al. confirmed that NSUN2i (Nsun2-i4) significantly reduces the m^5^C level in *SRSF6*, thereby impeding the nuclear export of SRSF6. Additionally, they found that treatment with NSUN2i combined with doxorubicin, cisplatin, or lenvatinib significantly inhibits tumor growth [100]. In addition, MY-1B, targeting NSUN2, also elicits potent anti-leukemic effects and demonstrates strong synergy with ferroptosis inducers, standard chemotherapy, and the BCL-2 inhibitor venetoclax [142]. Additionally, by highlighting key spatial configurations and physicochemical parameters, Fig. 6B provides critical insights that inform the rational design, optimization, and screening of future inhibitors targeting the same protein, thereby expanding the scope for innovative cancer therapies. The application of molecular docking in drug screening represents an effective strategy for identifying compounds that target NSUN catalytic sites. Collectively, these findings highlight NSUNs as promising novel targets for antitumor therapies, particularly in combination with immune checkpoint blockade for cancer immunotherapy.

In conclusion, NSUNs represent pivotal regulators and promising therapeutic targets in cancer prognosis and treatment. Further investigation into their precise regulatory mechanisms and biological functions, particularly focusing on structurally and functionally critical active sites, will provide a rational basis for the development of novel anticancer agents. Moreover, combination strategies—such as targeting NSUNs alongside conventional chemotherapy, immunotherapy, or pathway-specific inhibitors—may synergistically enhance treatment efficacy and overcome drug resistance. Advancing these efforts could pave the way for more personalized and effective anticancer therapies, ultimately improving patient outcomes.

### Summary and outlook

NSUN proteins are a key class of enzymes that catalyze RNA m^5^C modifications, exerting extensive regulatory effects on epitranscriptomic processes in both eukaryotic and prokaryotic systems. Accumulating evidence highlights the significant role of NSUNs in mammalian cells, particularly in the initiation and progression of cancer. This review provides a comprehensive analysis of the molecular mechanisms and biological functions of NSUN proteins in cancer, highlighting recent advancements in their application as biomarkers for tumor drug resistance and immune modulation, as well as the development of NSUN-targeted inhibitors as potential therapeutic agents.

At the molecular level, NSUNs promote tumor proliferation and metastasis by regulating RNA stability, splicing, processing, and translation. Dysregulation of NSUN proteins results from various mechanisms, including transcriptional control, post-translational modifications, and ncRNA-mediated regulation. Biologically, NSUNs influence processes such as programmed cell death, metabolic reprogramming in tumors, and angiogenesis. Additionally, NSUNs contribute to drug resistance through m^5^C modifications, affecting the efficacy of chemotherapeutic agents like cisplatin, oxaliplatin, and doxorubicin, as well as targeted therapies such as sorafenib, osimertinib, and olaparib.NSUNs, due to their essential role in cancer pathogenesis, have emerged as promising targets for therapeutic intervention, leading to the development of several inhibitors aimed at modulating NSUN activity. Despite the therapeutic potential of these inhibitors, research on NSUNs remains in its early stages, and the development and clinical translation of NSUN-targeted drugs are expected to encounter significant challenges. The therapeutic potential of NSUN-targeted inhibitors is also gaining attention. Future research aimed at elucidating NSUN mechanisms, developing targeted therapies, and conducting clinical trials will be instrumental in advancing cancer treatment strategies.

Although RNA m^5^C modifications can now be mapped at single-base resolution, their absolute quantification remains unresolved. Accurately quantifying m^5^C modifications and selectively introducing or deleting specific m^5^C sites could offer new insights into the functions and mechanisms of NSUN-mediated modifications. Bioinformatics currently plays a central role in studying m^5^C modifications, and artificial intelligence (AI) presents a promising approach to developing models that detect NSUN-mediated m^5^C-modified RNAs and identify their modification sites, thereby uncovering novel regulatory mechanisms in tumors. Moreover, AI may advance the use of m^5^C modifications as potential biomarkers or therapeutic targets in cancer, necessitating further exploration.
